# Dataset for an analysis of tourism and economic growth: A study of Sri Lanka

**DOI:** 10.1016/j.dib.2016.06.066

**Published:** 2016-07-06

**Authors:** Ronald Ravinesh Kumar, Peter Josef Stauvermann

**Affiliations:** a University of the South Pacific, Fiji; b Queensland University of Technology, Australia; c Changwon National University, South Korea

**Keywords:** Tourism demand analysis, Elasticity, Cointegration, Non-linear, Causality, Sri Lanka, Dataset

## Abstract

We use the sample from 1978 to 2014 for the paper (doi:10.1016/j.tmp.2016.05.005). The data on GDP at constant 2005 USD (US dollar), and the gross fixed capital formation at constant 2005 USD are extracted from the World Bank (2015). The labour stock which includes direct and indirect employment and the tourism receipts (in USD) are sourced from the Sri Lanka Tourism Development Authority (http://www.sltda.lk/statistics). Tourism receipts as a per cent of GDP is used to measure tourism demand. The capital stock data is computed using perpetual inventory method, where a depreciation rate of 8 per cent is assumed with the initial capital stock as 1.05 times the GDP of 1969 at constant 2005 USD. The output per worker and capital per worker is computed by dividing the GDP and capital stock by the labour stock, respectively.

**Specifications Table**

**Table:** 

Subject area	*Economics*
More specific subject area	*Tourism*
Type of data	*Annual data*
How data was acquired	*From the website of Sri Lanka Tourism Development Authority*
Data format	*Raw*
Experimental factors	*The data was handpicked from the PDF format and then appropriate transformations carried (see the paper for full details) for analysis*
Experimental features	1. *The data on visitor arrivals and tourism earnings are only available from the Sri Lanka Tourism Development Authority* (http://www.sltda.lk/statistics)
	2. *Other data sourced from World Bank*
Data source location	*Sri Lanka Tourism Development Authority, Sri Lanka*
	*World Bank Indicators and Global Development Finance*
Data accessibility	1. *Data is within this article and is publicly available from* http://sltda.lk/sites/default/files/Tourism%20Growth%201970%20-%202014.pdf 2. http://databank.worldbank.org/data/reports.aspx?source=world-development-indicators

**Value of the data**

• Data is useful for tourism and economic growth relationship analysis.
• Data is available in PDF format and is compiled by the local authorities.
• Data is publicly available, but with less awareness level.
• Other researchers may find the data useful for different types of analysis.

## 1 Data

The annual data extracted from the source is from 1970–2014. However, we use the sample from 1978 to 2014. Details are provided in the paper (doi:10.1016/j.tmp.2016.05.005).

## 2 Experimental design, materials and methods

The data is made available in PDF format by the Sri Lanka Tourism Development Authority. The data description and transformation are discussed in Kumar and Stauvermann [1]. (http://www.sltda.lk/statistics).
