# Supplementary material for: Dataset for an analysis of tourism and economic growth: A study of Sri Lanka
Source: Data Brief. 2016 Jul 6;8:723–5. doi: 10.1016/j.dib.2016.06.066 (PMC4950579; doi:10.1016/j.dib.2016.06.066)
Supplement: Supplementary file 1 — Supplementary material [file mmc1.doc]

23 June 2016

Managing Editor
Data in Brief

Dear Editor:

Re: Data in Brief for DOI: [10.1016/j.tmp.2016.05.005](http://dx.doi.org/10.1016/j.tmp.2016.05.005)

Thank you for the helpful comments. I have followed the comments and updated the data-in-brief document accordingly.

Additionally, I have enclosed the data spreadsheet.

On behalf of the co-author, I would like to declare that there is no conflict of interest. Moreover, the views expressed in the paper are ours and does not necessarily reflect the views of the affiliated organizations.

Please do not hesitate to contact us if further changes become necessary.

We take this opportunity to thank you once again.

Sincerely,

Ronald Ravinesh Kumar, PhD

Corresponding author.
